# Treatment of two Asiatic black bears (*Ursus thibetanus*) with severe injuries and their subsequent release into the wild: a case report

**DOI:** 10.1186/s12917-021-02834-9

**Published:** 2021-03-20

**Authors:** Dong-Hyuk Jeong, Kwangsik Jang, Jeong-Jin Yang, Joo-Yeul Choi, Seung-Hyo Lim, Seong-Chan Yeon, Kyung Mi Shim, Se Eun Kim, Seong Soo Kang

**Affiliations:** 1 Wildlife Medical Center, Korea National Park Service, Gurye, 57616 Republic of Korea; 2 Department of Veterinary Surgery and Biomaterial R&BD Center, College of Veterinary Medicine, Chonnam National University, Gwanju, 61186 Republic of Korea; 3 Department of Veterinary Clinical Sciences and Research Institute for Veterinary Science, College of Veterinary Medicine, Seoul National University, 1 Gwanak-ro, Gwanak-gu, Seoul, 08826 Republic of Korea

**Keywords:** Asiatic black bears, Wildlife, Injury, Amputation, Dual plate fixation, rhBMP-2, Rehabilitation, Re-introduction

## Abstract

**Background:**

The rehabilitation of injured wildlife and their subsequent release into the wild is a humane act as well as important in wildlife conservation. However, little is known about the animals’ fate after release. Therefore, to address these uncertainties, it is essential to adequately describe how the injured animals were treated and managed before releasing into the wild; moreover, post-release monitoring should also be performed. Herein, we document for the first time the process of rescue, surgery, and rehabilitation of severely injured Asiatic black bears (*Ursus thibetanus*; endangered species in South Korea) and their fate after returning to the wild.

**Case presentation:**

A six-year-old female (bear-01) and a three-year-old male (bear-02) bears were injured by an illegal snare and collision with a bus, respectively. Bear-01 had broad muscle necrosis and ruptures from the snared ankle on the right thoracic limb, with myiasis, and elbow disarticulation was performed. In bear-02, a non-reducible comminuted fracture of the left humerus was confirmed radiologically, and the operation was performed by using dual plate fixation with hydroxyapatite and recombinant human bone morphogenetic protein-2. The bear-01 and -02 were completely healed approximately 30 and 60 days after surgery, respectively. After that, they underwent rehabilitation for 8 and 25 days, respectively, in an outdoor enclosure similar to their natural habitat. Bear-01 and -02 were released into the wild after 45 and 99 days after surgery, respectively, and their mean daily movement distance during the first 30 days after releasing was 2.9 ± 2.1 and 1.3 ± 1.6 km, respectively. The annual mean 95% Kernel home-range size of bear-01 and bear-02 was 265.8 and 486.9 km^2^, respectively. They hibernated every winter, gained weight, gave birth to cubs (bear-01), were not found to have any abnormalities in the veterinary tests, and were not involved in any conflicts with humans after returning to the wild.

**Conclusions:**

Bears without one leg or those with dual plates could adapt well in their natural habitat, which shows that our surgical and post-operative treatments were effective. Additionally, minimizing human contact and observing/evaluating behavior during the rehabilitation is essential in reducing human-bear conflicts after release.

## Background

The rehabilitation and returning to the wild of injured or ill wildlife is a humane act as well as being an important part of wildlife conservation; however, the release of injured animals back into the wild is often controversial [1–3]. Since wildlife rehabilitation and release are a blend of veterinary medicine, natural history, ecology, and animal behavior [4], they are occasionally performed without adequate data on animal husbandry, nutrition and restraint techniques, disease susceptibility, biomedical parameters, and specific medical and surgical techniques [5–7]. In addition, little is known about the animals’ fate after release, their impact on the local receiving population, the outcome of their health, or whether their release into the wild was successful. Population-level risks may also occur if the released animals are more likely to become involved in human-wildlife conflicts or to spread diseases [3, 8]. Therefore, to address these uncertainties, post-release monitoring should be conducted to clarify the feasibility of this approach for wildlife conservation [9]. Beecham et al. [10] reported that captive-reared bears released to the wild met the requirements for success concerning survival rates, human conflict levels, and reproduction in the wild. However, all bears accepted into captive-rearing facilities in the study were orphaned cubs without severe injuries. The combination of a series of treatment, rehabilitation, release, and post-release monitoring of adult bears with severe injuries has been rarely been reported.

The Asiatic black bear (*Ursus thibetanus*; ABB) is a vulnerable species according to the Red List by the International Union for Conservation of Nature [11], and a reintroduction project to establish a self-sustaining ABB population was initiated in 2002 in the Republic of Korea [12]. Highlights of the project include the introduction and releasing into the wild of ABB from China, North Korea and Russia, research and monitoring of these individuals, and implementation of public awareness-raising activities to protect species [12]. The number of bears, which was only about 5 at the beginning of the project, has increased to 69 as of 2020, which is considered a very successful restoration project [13]. We focused on 2 severely injured ABB that were rehabilitated and released into the wild in 2017 and 2018. The study areas were Jirisan National Park, Gayasan National Park, Mt Sudo, and its surrounding areas where the restoration project of Asiatic black bears is underway (35°12′40′′ – 35°56′ 04′′ N and 127°27′ 20′′ – 128°08′18′′ E). In this context, our releases were intended to reinforce the local ABB population, adding 2 individuals that might become breeders into the resident population. In addition, a series of emergency rescue, treatment, rehabilitation, release, and post-release monitoring conducted in our cases might represent one of the most active wildlife conservation efforts. Herein, we documented the process of rescue, surgery, rehabilitation of severely injured ABBs which were introduced from Russia to Jirisan National Park in South Korea in 2012 and 2016, and their fate after returning to the wild. All work reported in this study was carried out under license from the Korea National Park Service (permits: SRTI-1727, 2783, 2924, and 3301).

## Case presentation

### Case 1

Six-year-old female ABB (bear-01) had been monitored through radio-tracking since the first release at the age of 1 year for the restoration of endangered ABB in South Korea, was found severely injured by an illegal snare trap to catch wild boars on September 08, 2017.

**Fig. 1:**
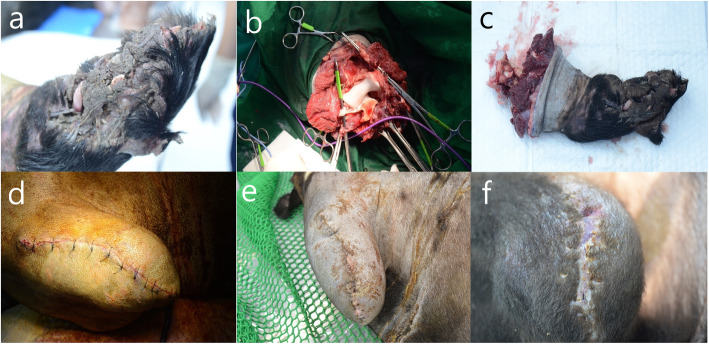
The amputation of the elbow joint in the Asiatic black bear (bear-01). **a**, before the surgery; **b**, exposed trochlea and capitulum of right humerus; **c**, amputated right thoracic limb; **d**, immediately after surgery; **e**, 5 days after surgery; **f**, 37 days after surgery

The bear was held in the trap for less than 2 days (2 days ago, it was confirmed by radio tracking that the bear was in a place other than the trap location), and it was immobilized with zolazepam-tiletamine (2 mg/kg; Zoletil 50®, Virbac, Carros, France) and medetomidine (0.04 mg/kg; Domitor®, Pfizer, New York, NY, USA) using a CO_2_-powered gun (Dan-inject, Børkop, Denmark) for removal of the trap. Then, the bear was transported to the Wildlife Medical Center of Korea National Park Service for clinical assistance and rehabilitation, and there was no supplementary injection of anesthetics during the transportation. The animal weighed 104 kg at capture, and the examination revealed broad muscle necrosis and rupture of the snared ankle on the right thoracic limb with myiasis (Fig. 1a); blood tests showed increased aspartate aminotransferase (AST) and creatine phosphokinase. Thus, surgical amputation of the injured limb was required and we operated on September 09, 2017. The animal was immobilized with the standard method prescribed for ABB by Jeong et al. [14]. Briefly, intramuscular injections of zolazepam-tiletamine (2 mg/kg; Zoletil 50®) and medetomidine (0.04 mg/kg; Domitor®) was administered from a distance of 5 m using a CO_2_-powered gun (Dan-inject); anesthesia was maintained with isoflurane (Terrell®, Piramal Critical Care, Bethlehem, PA, USA) in 100% oxygen (4 L/min) administered via a 10-mm endotracheal tube intubation in a circle rebreathing system. After intubation, the bear received injections of glycopyrrolate (0.01 mg/kg, IM; Mobinul®, Myungmoon Pharmaceutical Co., Ltd., Seoul, South Korea), cefovecin sodium (8 mg/kg, SC; Convenia®, Zoetis, Parsippany, NJ, USA), tramadol (2 mg/kg, IM; Humedix Tramadol HCl INJ.®, Huons Co., Ltd., Seongnam, South Korea), and meloxicam (0.2 mg/kg, SC; Metacam®, Boehringer Ingelheim, St. Joseph, MO, USA). Lactated Ringer’s solution (Hartmann’s Sol., Daihan Pharmaceutical Co., Ltd., Seoul, South Korea) was administered intravenously at a rate of 10 ml/kg per hr. In addition, vital signs such as heart rate, respiratory rate, peripheral capillary oxygen saturation (SpO_2_), rectal temperature, non-invasive blood pressure, and end-tidal carbon dioxide partial pressure (ETCO_2_) were monitored with a dedicated monitor (Datex-Ohmeda S/5, GE Healthcare Life Science, Helsinki, Finland).

The elbow disarticulation was performed and the procedure followed the method described by Winkler [15] and Morrey [16], with some modifications (Fig. 1b-d). Briefly, equal anterior and posterior skin flaps were created, beginning at the level of the humeral epicondyles. The incisions are extended distally, 5 cm distal to the tip of the olecranon posteriorly, and down to a point just distal to the insertion of the biceps tendon anteriorly. The lacertus fibrosus was divided and all the pronator attachments from the medial epicondyle were released. Then, the radial and ulnar nerves were transected and allowed to retract into the proximal wound. The large vessels were doubly ligated with 3–0 polyethylene terephthalate (Ti-CRON Polyester sutures, Covidien, Dublin, Ireland) and transected by an electro-thermal vessel sealing device (EVSD; LigaSure Max, Covidien, Dublin, Ireland); the small vessels were transected using the device alone, without ligation. The anterior, radiohumeral, and ulnohumeral capsules were divided, and trochlea and capitulum of the humerus were exposed. Then, the radius was disarticulated; however, it was difficult to disarticulate the ulna. Therefore, we performed an ostectomy of the ulna at the level of the trochlea, retaining the olecranon of the ulna, using an electric saw (BH 100S, IMEDICOM, Gunpo, South Korea). The posterior flap was moved medially and sutured continuously to the soft tissues of the medial epicondyle with 2–0 polyglyconate (Maxon®, Covidien, Dublin, Ireland). The humerus was closed with a flap of the brachialis, biceps, and triceps, and the skin was closed with a simple interrupted suture of 1–0 nylon (Blue nylon NB125, Ailee, Busan, Korea) and skin stapler (Appose ULC 35 W, Medtronic, Minneapolis, MN, USA). After transportation to the rehabilitation room, 0.2 mg/kg atipamezole (Antisedan®, Pfizer, New York, NY, USA) was injected intravenously, and the animal recovered from general anesthesia after a duration of 337 min uneventfully in a metal holding cage (2.2 × 0.8 × 1.2 m). The total surgery time was 268 min, and all parameters of intraoperative anesthetic evaluation were within the normal ranges (mean respiratory rate, 13.9 ± 1.7 breaths/min; mean heart rate, 54.4 ± 8.1 beats/min; mean rectal temperature, 35.9 ± 0.7 °C; mean blood pressure, 91.1 ± 11.8 mmHg; mean SpO_2_, 97.9 ± 4.5%; mean ETCO_2_, 30.5 ± 1.3%). The bear-01 stayed in the holding cage for 22 days and was visited twice daily for approximately 5 min for feeding (the first 5 days after the surgery) veterinary prescription diet (Recovery, Royal Canin, Aimargues, France) and fruits with honey; from days 6–22, acorns, chestnuts, fruits, vegetables, sweet potatoes, and commercial feed (Omnivore Diet Dry, ZuPreem, Mission, TX, USA), and medication (0.1 mg/kg meloxicam with food once a day for 14 days orally) were administered. No post-operative complications were observed during the stay.

On October 01, 2017, the bear-01 was moved to an indoor room (3 × 4 × 3 m; tiled floors and walls; direct sunlight via a 1 m × 1.4 m window on the roof) for light exercise and recovery and stayed there for 15 days. On the 37th day after the surgery, the surgical site was examined and the skin staples were removed under general anesthesia (Fig. 1f). The examination confirmed that the surgical site had recovered completely; on the same day, we moved the animal for rehabilitation to an outdoor enclosure (5489 m^2^), established a closed-circuit television system (CCTV; IDR-H4032, IDIS Korea, Daejeon, South Korea) in the forest, which was similar to the bear’s natural habitat. While it was there for 8 days, we observed its movement and behavior through CCTV without any direct contact and confirmed that the bear climbed up and down the trees without difficulty and found its natural foods, and ate them in the enclosure. To see how the bear reacted to humans, two people went into the enclosure with the bear’s favorite foods (apple, honey, fish and chestnut); immediately after their entrance, the bear avoided the people and moved away from them. Additionally, we established an portable electric fence (3 × 3 m) in the enclosure and placed some foods inside the fence (Fig. 4a) to see how the bear reacted to the fence; the fence was non-invasive and safe for bears weighing over 100 kg because the average voltage of the electric fence was only 4 kW and it passes current for 0.3 s once a second. As a result, the bear approached the fence and tried to enter during the night on three occasions, but received an electric shock. Subsequently, the bear roamed around it for about 5 min, but the bear did not pass beyond the fence (Fig. 4b). Considering these behaviors (avoidance of humans and recognizing the electric fence), activities, and the bear’s general health condition, we concluded that the bear could be returned to its natural habitat. Consequently, we released the bear, fitted with a radio transmitter (M3620, ATS, Isanti, MN, USA), into Jirisan National Park on October 24, 2017 (45 days after surgery), and radio-tracked it every day for about 2 years through a triangulation method [17] and obtained 435 locations. In addition, we accompanied a field survey (camera trapping and hibernation site survey) as well. We estimated daily movement distances (DMD) as the straight-line distances (km) between two consecutive days and analyzed the area covered by the bear using minimum convex polygons (MCP) with 95% and kernel with 95 and 50% of locations using a geographic information system (ArcGIS v.10.1, Environmental Systems Research Institute, Inc., Redlands, CA, USA). Telemetry error was estimated by comparing the estimated locations of nine test transmitters by five experts with more than 6 years of radio-tracking experience to their actual locations. The locations of the test transmitters were changed once, thus generating 18 different locations. Consequently, 90 radio-locations for telemetry error (5 people × 18 locations) were obtained and the average telemetry error was 44.7 ± 39.6 m. The mean DMD, 95% MCP, 95 and 50% kernel home ranges for 30 days between the day they were released and the day before hibernation (November 25, 2017; the hibernating day was defined as the first day of the period in which the location change within 100 m lasts continuously for 5 days or more) were 1.3 ± 1.6 km, 9.4 km^2^, 56.9 km^2^, and 10.5 km^2^, respectively (Figure or Table 1). On the March 28, 2018, we surveyed the den site and observed bear-01, which was playing with two cubs (Fig. 4d, e). Then, they stayed there until May 15, 2018, mean DMD, 95% MCP, 95 and 50% kernel home ranges were 1.6 ± 1.8 km, 41.7 km^2^, 138.7 km^2^, and 30.5 km^2^ in summer (June to August), and those in autumn (September to November) 2018 were 2.1 ± 2.2 km, 56.9 km^2^, 189.9 km^2^, and 53.5 km^2^, respectively (Figure or Table 1). Between January 15 and February 20, 2019, the bear-01 hibernated, and it was re-captured on the 10th of August 2019 to exchange the transmitter by a culvert trap. When bear-01 was captured, it weighed 105 kg, and through camera trapping and field surveys we could not find any evidence that the bear was living with the yearlings. The blood sample was collected from the jugular vein, and all blood chemistry and complete blood cell count (CBC) test values were within normal ranges. The mean DMD, seasonal, and annual home ranges in 2019 are shown in Table 1. On the 18th of May 2020, we confirmed bear-01 with two newborn cubs through camera trapping (Fig. 4f).

**Table 1:** The summary of rescue, treatment, rehabilitation, release, and post-release monitoring in two severely injured Asiatic black bears (*Ursus thibetanus*) in the Republic of Korea

***Bear-01***						
Sex	Age	Rescue	Surgery	Recovery	Rehabilitation	Release
Female	6 years	104 kg, broad muscle necrosis and ruptures of the snared ankle on the right thoracic limb with myiasis (8/9/2017)	The elbow disarticulation (9/9/2017)	1. Small cage (22 days) 2. Indoor room (15 days) Completely healed around 30 days after surgery	Preparation for returning to nature and behavior assessment in outdoor enclosure (8 days)	Jirisan NP (45 days after surgery; 24/10/2017)
Post-release monitoring (radio-tracking and field survey)						
DMD (km)		HR for 95% MCP (km^2^)	HR for 95% Kernel (km^2^)	HR for 50% Kernel (km^2^)		Note
The first 30 days: 1.3 ± 1.6 Summer 2018: 1.6 ± 1.8 Autumn 2018: 2.1 ± 2.2 Spring 2019: 1.6 ± 2.3 Summer 2019: 2.8 ± 2.4 Autumn 2019: 1.1 ± 1.6		The first 30 days: 9.4 Summer 2018: 41.7 Autumn 2018: 56.9 Spring 2019: 50.1 Summer 2019: 72.5 Autumn 2019: 82.4 Annual 2019: 68.3	The first 30 days: 56.9 Summer 2018: 138.7 Autumn 2018: 189.9 Spring 2019: 229.6 Summer 2019: 250.1 Autumn 2019: 317.7 Annual 2019: 265.8	The first 30 days: 10.5 Summer 2018: 30.5 Autumn 2018: 53.3 Spring 2019: 64.2 Summer 2019: 67.5 Autumn 2019: 71.6 Annual 2019: 67.8		Parturition of four cubs after releasing into the wild (two in 2018, two in 2020; confirmed by unmanned camera and field survey) 105 kg (confirmed by capturing it with a culvert trap, 10/8/2019)
***Bear-02***						
Sex	Age	Rescue	Surgery	Recovery	Rehabilitation	Release
Male	3 years	110 kg, comminuted fracture of left humerus (8/9/2017)	Fixation of two locking compression plates with screws + autologous bone chips + rhBMP-2 (17/5/2018)	1. Small cage (48 days) 2. Indoor room (26 days) Completely healed around 60 days after surgery	Preparation for returning to nature and behavior assessment in outdoor enclosure (25 days)	Jirisan NP (99 days after surgery; 26/8/2018)
Post-release monitoring (radio-tracking and field survey)						
DMD (km)		HR for 95% MCP (km^2^)	HR for 95% Kernel (km^2^)	HR for 50% Kernel (km^2^)		Note
The first 30 days: 2.9 ± 2.1 Autumn 2018: 1.9 ± 1.7 Spring 2019: 1.4 ± 2.1 Summer 2019: 3.7 ± 2.5 Autumn 2019: 0.8 ± 1.0		The first 30 days: 36.9 Autumn 2018: 106.7 Spring 2019: 95.6 Summer 2019: 419.6 Autumn 2019: 24.6 Annual 2019: 179.9	The first 30 days: 170.6 Autumn 2018: 215.8 Spring 2019: 247.1 Summer 2019: 1131.1 Autumn 2019: 82.5 Annual 2019: 486.9	The first 30 days: 40.5 Autumn 2018: 59.2 Spring 2019: 53.4 Summer 2019: 213.1 Autumn 2019: 19.2 Annual 2019: 95.2		143 kg (confirmed by winter den capturing, 26/2/2018) 165 kg (confirmed by winter den capturing, 27/2/2019)

Abbreviations: *NP* National Park; *DMD* daily movement distance as the straight-line distance between consecutive 2 days; *HR* home range; *MCP* minimum convex polygons; rhBMP-2, synthetic hydroxyapatites, and recombinant human bone morphogenetic protein-2. Spring, summer, and autumn are March to June, June to August, and September to November, respectively. The DMD was presented as mean ± standard deviation

### Case 2

**Fig. 2:**
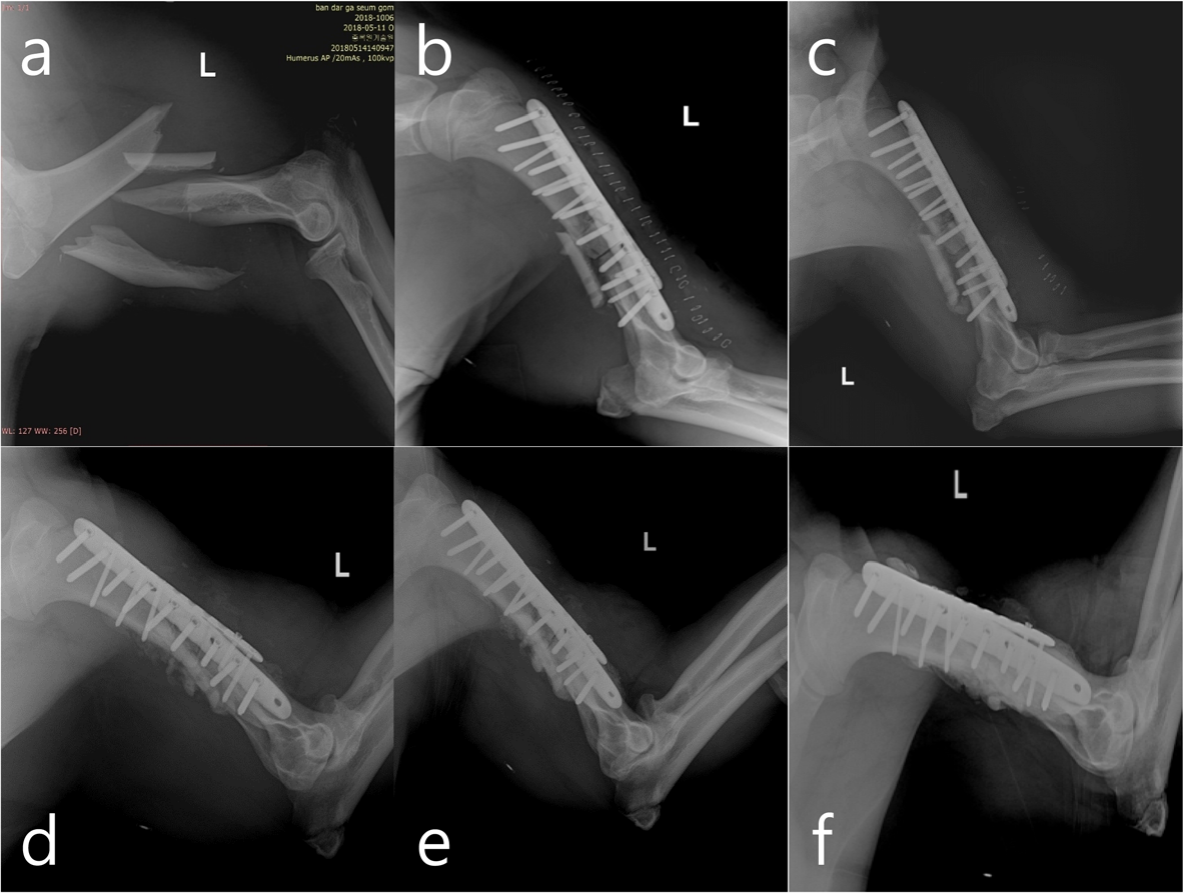
Lateral radiographs of the humerus in the Asiatic black bear (bear-02). **a**, comminuted fracture before surgery; **b**, immediately after surgery; **c**, 21 days after surgery; **d**, 34 days after surgery; **e**, 48 days after surgery; **f**, 62 days after surgery

**Fig. 3:**
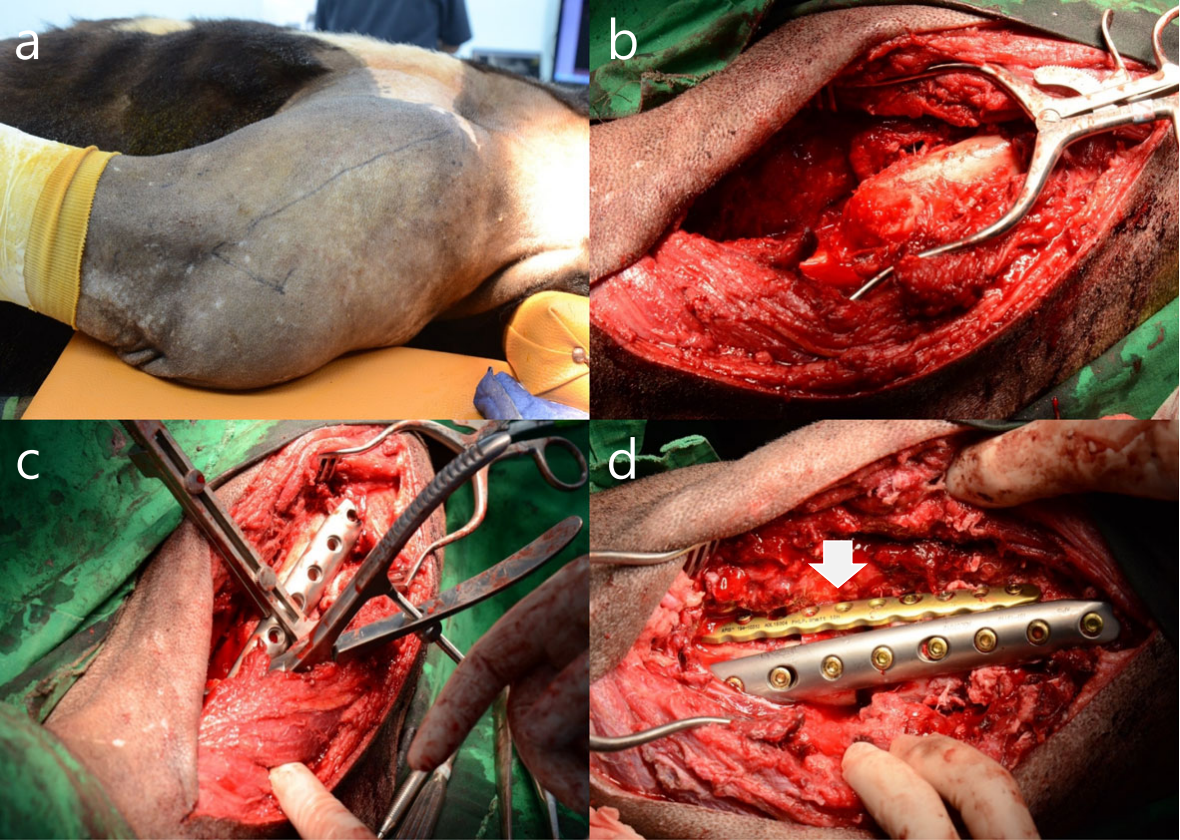
The treatment of humoral fracture using dual plate fixation in the Asiatic black bear (bear-02). **a**, lateral approach and incision line; **b**, fractured humerus; **c**, lateral application of 10 holes’ 5.0 locking compression plate with 10 screws (32 mm, 1; 34 mm, 1; 36 mm, 3; 38 mm, 1; 40 mm, 2; 48 mm, 2); **d**, anterior application of 4.0 locking compression plate with 5 screws (26 mm, 36 mm, 38 mm, 40 mm and 42 mm), white arrow

**Fig. 4:**
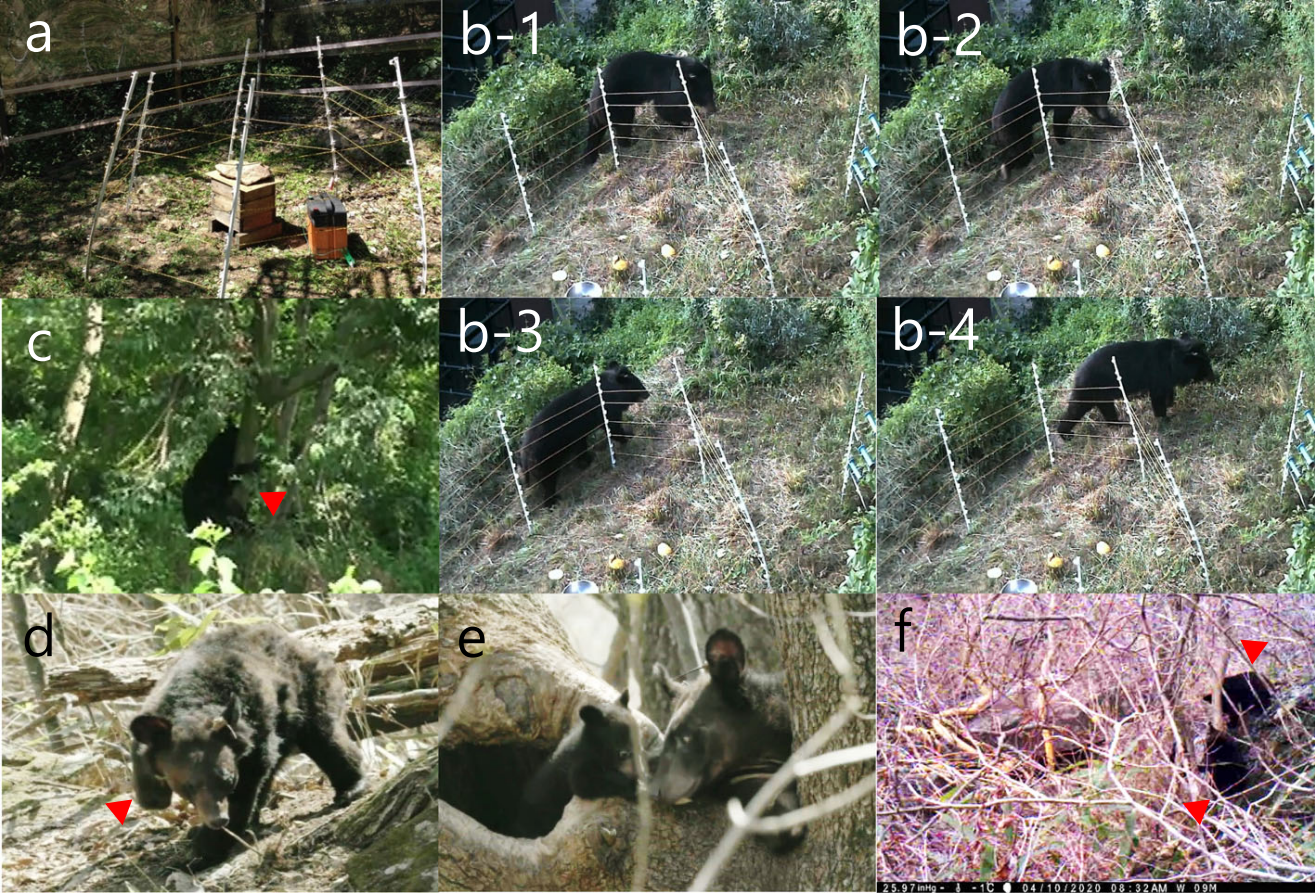
Rehabilitation at the outdoor enclosure before releasing and post-releasing monitoring of two Asiatic black bears. **a**, portable electric fence with honey in the wooden box; **b-1, − 2, − 3, and − 4**, the bear-02 (plates implanted) that passed by without going inside an electric fence with food during the rehabilitation period; **c**, the bear-02 that used all four legs to climb trees (red arrow); **d**, the bear-01 with an amputated right limb (red arrow) from the first hibernation after returning to nature; **e**, the bear-01 with a cub that gave birth during its first hibernation since returning to nature; **f**, two cubs that gave birth during the third hibernation of bear-01 after returning to nature. The photos were taken by a smart phone camera or unmanned camera

A three-year-old male bear (bear-02), which was managed by the reintroduction program of ABB, was rescued from a traffic accident on the May 11, 2018 (bear-02 moved to the mountains after the collision with a bus on the 5th of May and we were able to capture him on the 11th of May). When captured, the animal weighed 110 kg and seemed to have a fracture of the left humerus. This bear was transferred to the same veterinary team and a non-reducible comminuted fracture of the left humerus was confirmed radiographically (Fig. 2a). Blood examinations revealed increased white blood cells, AST, and C-reactive protein. On the 17th of May (12 days after the accident), preoperative radiographs of both the injured and the contralateral humerus were obtained. From these radiographs, a plate of the appropriate length was selected in an attempt to span the entire length of the humerus. Immobilization and monitoring of vital signs were followed by the same procedures used for bear-01. Bear-02 was placed in a supine position and we approached laterally for the surgery. A longitudinal skin incision was made from the center of the deltoid insertion to the lateral epicondyle, and the lateral head of the triceps was exposed by the insertion of the brachial fascia. The humerus was exposed by elevation of the brachialis and triceps from the lateral intramuscular septum. We tried to remove a bone fragment that was lodged in the deep medial head of the triceps and repair the alignment of the humerus; however, it was impossible because of the exuberant granulations and muscular contraction. Thus, we only cut the sharp edge of the broken humerus using an electric surgical saw (BH 100S, IMEDICOM, Gunpo, South Korea) without removal of the bone fragment and then we repaired the alignment of the humerus. Then, a 10 holes’ 5.0 locking compression plate (LCP; APIS 157–18,110, TDM, Inc., Gwangju, South Korea) with 10 locking head screws (32 mm, 1; 34 mm, 1; 36 mm, 3; 38 mm, 1; 40 mm, 2; 48 mm, 2) and a 10 holes’ 4.0 LCP (APIS 194–10,310, TDM, Inc., Gwangju, South Korea) with 5 locking head screws (26 mm, 36 mm, 38 mm, 40 mm and 42 mm) were used for double-plate fixation (anterior and lateral surfaces of the humerus), respectively (Fig. 3). In addition, autologous bone chips (the pieces cut from the edge of fractured humerus by surgical saw were crushed in a mortar), synthetic hydroxyapatite, and recombinant human bone morphogenetic protein-2 (rhBMP-2; NOVISIS®, CGBIO, Inc., Seungnam, South Korea) were mixed according to the manufacturer’s manual and it was filled in the bone defect areas. The muscles and soft tissue were sutured continuously with 2–0 polyglyconate (Covidien Maxon sutures, Covidien, Dublin, Ireland) and the skin was closed with a skin stapler (Appose ULC 35 W, Medtronic, Minneapolis, MN, USA). Total surgery time was 405 min and the duration of anesthesia was 479 min. All parameters for anesthetic evaluation during the surgery were within normal ranges (mean respiratory rate, 13.5 ± 2.1 breaths/min; mean heart rate, 78.6 ± 25.6 beats/min; mean rectal temperature, 36.4 ± 0.5 °C; mean blood pressure, 113.1 ± 23.8 mmHg; mean SpO_2_, 97.9 ± 4.5%; mean ETCO_2_, 35.1 ± 4.7%). Medication during and after the surgery and the post-operative management were the same as those used for bear-01, except as indicated below. Postoperatively, bear-02 recovered in a metal holding cage (220 × 80 × 120 cm) for 48 days and radiographs were acquired on the 21st, 34th, and 48th days after the surgery (Fig. 2). During this period, we confirmed the formation of a callus and an increasing density of the fracture line. Thus, on the 5th of July (48 days after the surgery), we moved bear-02 to the indoor room (3 × 4 × 3 m) for light exercise, where it recovered for 26 days. While staying in the indoor room, we acquired radiographs (on the 62nd day after the surgery) and confirmed complete healing of the fracture areas (Fig. 2). Bear-02 showed no abnormal walking, and we determined that the bear needed more free and vigorous exercise. Consequently, on the 2nd of August (74 days after surgery), we moved the animal to the outdoor enclosure in the forest for rehabilitation, and the animal stayed there another 25 days. During its stay there, the bear walked up and down the slope naturally and climbed up and down a tree easily (Fig. 4c). Additionally, the bear’s behavior was evaluated in the same way by the same observers for bear-01, and bear-02 showed similar behaviors as bear-01 (active human avoidance, does not try to get through the electric fence to enter the inside where the food is (Fig. 4b) after imprinting of the electric fence). Thus, on the 27th of August, 2018 (99 days after the surgery), we released it, after fitting a radio transmitter (M3620, ATS, Isanti, MN, USA) into Mt. Sudo, Kyoungbuk province; post-release monitoring and analysis were performed as that for bear-01. During the study, we obtained 465 locations and mean DMD, 95% MCP, 95 and 50% kernel home ranges for the first 30 days after releasing were 2.9 ± 2.1 km, 36.9 km^2^, 170.6 km^2^, and 40.5 km^2^, respectively (Table 1). Additionally, those in autumn 2018 were 1.9 ± 1.7 km, 106.7 km^2^, 215.8 km^2^, and 59.2 km^2^, respectively (Table 1), and bear-02 hibernated from the 27th of December 2018 until the 3rd of March 2019. During hibernation, we visited at its den site by radio-tracking and immobilized the bear there using a dart gun to replace the transmitter because of the battery’s abnormal exhaustion, and its weight was 143 kg. The mean DMD, seasonal, and annual home ranges in 2019 are shown in Table 1, and when bear-02 was captured, on the 27th of February 2020, during the 2nd hibernation after returning to nature, its weight was 165 kg. The veterinary examination including blood chemistry and CBC in those two captures found no abnormal findings.

## Discussion and conclusions

Studies have previously reported orphaned bears reared in captivity and their release into the wild [10, 18–20], but there is little information about the treatment, rehabilitation, and post-release monitoring of seriously injured bears over 3 years of age. Thus, herein we described in detail these processes; our methodologies and results will be a great reference for those performing related activities, such as wildlife veterinarians, ecologists, officers, and rehabilitators.

The elbow disarticulation was performed in bear-01 because if a transhumeral amputation or scapulectomy was conducted, the surgical site could be broadened owing to the considerably thick upper limb and wide shoulder areas of the bear. Consequently, this might increase the surgery time and make post-operative management difficult. In addition, the advantage of elbow disarticulation in the bear was that the animal could use its upper limb after surgery, which could help it climb trees or balance on them, and this was confirmed as we observed that indeed the bear was climbing the tree using its amputated right limb. Additionally, there was little blood loss from vascular cutting during surgery, and there were no complications such as bleeding and formation of a seroma during the recovery period. This shows that using EVSD together rather than double ligation of vessels can help minimize bleeding in an amputation. Furthermore, the operation time was shortened by using EVSD without ligation before cutting the small vessels, and this is considered to be a very efficient method for long-term surgery of large animals.

In case 1, the bear gave birth to two cubs after returning to its natural habitat despite being under a prolonged period of anesthesia and surgery. Considering that the day of the surgery and the day it was released into the wild were in late August and late October, respectively, the bear might have already had some fertilized eggs at the time of the operation; the ABBs in Korea only mate between May to August [21] and implantation in brown bears occurs in late November to early December [22, 23]. It was reported that a high concentration of isoflurane negatively affects in vitro fertilization of inseminated oocytes in mice [24], but its correlation between anesthesia and development of a fertilized egg in bears is unknown. In case-01, we have confirmed the birth of two healthy cubs of a female bear that had fertilized eggs under prolonged anesthesia, and this supports the fact that the anesthesia protocol using zolazepam-tiletamine-isoflurane was safe in both ABB and its fertilized eggs.

In case-02, we repaired the comminuted fracture of the left humerus in a bear and this is the first report on the treatment of humral fracture using dual plate fixation in bears. The unique and complex shape of the humerus makes fracture repairs challenging and the lateral aspect of the humerus is widely accepted as the tension side of the bone [25]. Similarly, in this case, large and thick muscles were attached to the humerus and there were strong muscle contractions and torsion with exuberant granulations. Moreover, since the bear was one of the animals that use their forelimbs frequently and are important for their survival, it was important to fix the bone fragments firmly during the recovery, and the repaired forelimb should be able to withstand the heavyweight of the bear itself, as well as the powerful forces that occur when the bear uses its forelimb. Thus, we used a locking compression plate (LCP), because it has advantages in comminuted fractures such as preservation of the blood supply by reducing the contact with the bone [26, 27], improvement of callus formation [28, 29] and no need for exact contouring of the plate which saves time and decreases the chances for loss of reduction [30]. Additionally, the LCP construction has four times more strength compared to conventional plating [31], but it does not depend on the screw purchase in the bone because the head of the screw fixes to the plate and as a result, is more advantageous in comminuted fractures [29, 32, 33]. Furthermore, we filled the mixture of autologous bone chips, synthetic hydroxyapatite, and rhBMP-2 in the bone defect areas. This trial would be helpful to accelerate bone formation and regeneration of defective bone tissues in the bear as well, although it was not clear how much it worked in this case. Because the osteogenic potential of BMP has been widely accepted and the suitability of hydroxyapatite as its carrier in rats, dogs, and cats including humans [34–38].

It was reported that 65% of long-bone fractured horses, including the humerus, showed complications after fracture repairing surgery [39]. In our case, no post-operative complications were observed during the stay during captivity and even after returning to nature. This successful treatment was possible not only because of the use of LCP and rhBMP-2 but also because the plates were applied dually and the exercise was restricted in a small cage after surgery. Although the application of a dual plate is not common in veterinary practice, Crawford and Fretz [39] reported that surgery with a dual plate had a higher success rate in the treatment of long-bone fractured horses and cattle, and Bologna et al. [40] showed that the use of a dual plate showed a higher bone union rate than an operation with a single plate in humans.

For a seriously injured bear, the survival and recovery of its health are the most important proposition, but the most important thing to return to the wild after recovery of health is to behave like normal wild individuals and to show a tendency to avoid humans. Because any alterations of their behavior that make the bear more dependent on anthropogenic food sources or more exposed to humans will likely result in reduced survival [41]. Captive experience, in general, could negatively influence a bear’s natural behavior in the wild and several studies reported unsuccessful reintroductions of captive bears [41–43]. That means it is difficult to maintain the wildness and natural behavior of bears in captivity. There is a limit to evaluating the home-range size of our released bears because a reference to the average home range size of ABBs in temperate forest areas is very rare, but comparing to the home-range size of American black bears in Washington [44] and Florida [45] in the U.S. (annual mean 95% kernel home-range size of female and male in our cases, Washington and Florida were 265.8 km^2^ and 486.9 km^2^ vs 24.1 ± 5.4 km^2^ and 96.6 ± 26.5 km^2^ vs 24.2 ± 3.6 km^2^ (female), respectively), bears in our cases seem to have a wide home-range and lots of movements despite the amputation of the leg or the implantation of the plates. This indicates that seriously injured bears fully recovered, but the question remains whether they were really well adapted to nature after releasing. Because home range size in bears subsequently varied from 2.4 km^2^ to 295 km^2^ [46–50] and could be influenced by several factors, including the distribution and abundance of resources, population density, degree of habitat fragmentation and associated anthropogenic effects [44, 45], so in addition to the home range, another indicator such as hibernation, mating, weight, disease infection and conflict with humans should be evaluated comprehensively to determine the natural adaptation after releasing. In our cases, the bears hibernated every winter, increased weight, gave birth to cubs (bear-01), were not found to have any abnormalities in the veterinary tests, and made no conflict issues with humans after releasing them into the wild. These facts support that our released bears adapted well to nature and that the way we took care of and rehabilitated the bears helped them return to nature.

A single bear’s emergency rescue has a great impact on the restoration of the population in isolated habitats, not just on the animal at a humanitarian level. The rescue and returning to the nature of bear-01 had the effect of saving five due to the birth of a total of four cubs twice after release. Also, bear-02 allowed the preservation of a male that can participate in breeding, thereby contributing to continued reproduction in and out of the habitat (1. natural reproduction within a population; 2. when recaptured, semen collected at the site is frozen and can be used for assisted reproductive technology, such as artificial insemination and in vitro fertilization) [51, 52]. In the long term, these two individuals have a preservative effect of at least 6 (bear-01 and its 4 cubs, bear-02) to about 15 individuals (Assumption 1, 10 years’ breeding period; 2, the male mate with at least one female per year; 3, they are not defeated by the breeding competition; 4, female give birth every other year and have 2 cubs; 5, the survival rate of the cubs is 50%), and if individuals born by artificial insemination using semen of bear-02 are included, the preservative effect would be greater.

In conclusion, we showed the processes and results of a successful treatment, rehabilitation, and return to the wild of severely injured ABBs. We confirmed our surgical methods and post-operative managements were effective and even bears without one leg or with dual plates can adapt well in nature. Additionally, minimizing human contact and observing/evaluating behavior during the rehabilitation process is essential to reducing human-bear conflicts after release. Successful restoration of endangered species in isolated habitats requires that the project includes a team that can be fielded, as well as a specialized veterinary team, and continuous monitoring of the populations after release.

## Data Availability

Not applicable.

## Abbreviations

ABB: Asiatic black bear
AST: Aspartate aminotransferase
CBC: Complete blood cell count
CCTV: Closed-circuit television system
DMD: Daily movement distances
ETCO_2_: End-tidal carbon dioxide partial pressure
EVSD: Electro-thermal vessel sealing device
HR: Home range
LCP: Locking compression plate
MCP: Minimum convex polygons
NP: National Park
rhBMP-2: Recombinant human bone morphogenetic protein-2
SpO_2_: Peripheral capillary oxygen saturation
